# The Impact of Formulation on the Content of Phenolic Compounds in Snacks Enriched with *Dracocephalum moldavica* L. Seeds: Introduction to Receiving a New Functional Food Product

**DOI:** 10.3390/molecules26051245

**Published:** 2021-02-25

**Authors:** Tomasz Oniszczuk, Kamila Kasprzak-Drozd, Marta Olech, Agnieszka Wójtowicz, Renata Nowak, Robert Rusinek, Jarosław Szponar, Maciej Combrzyński, Anna Oniszczuk

**Affiliations:** 1 Department of Thermal Technology and Food Process Engineering, University of Life Sciences in Lublin, Głęboka 31, 20-612 Lublin, Poland; agnieszka.wojtowicz@up.lublin.pl (A.W.); maciej.combrzynski@up.lublin.pl (M.C.); 2 Department of Inorganic Chemistry, Medical University of Lublin, Chodźki 4a, 20-093 Lublin, Poland; 3 Department of Pharmaceutical Botany, Medical University of Lublin, Chodźki 1, 20-093 Lublin, Poland; renata.nowak@umlub.pl; 4 Institute of Agrophysics, Polish Academy of Sciences, Doświadczalna 4, 20-290 Lublin, Poland; r.rusinek@ipan.lublin.pl; 5 Toxicology Clinic, Clinical Department of Toxicology and Cardiology, Medical University of Lublin, Stefan Wyszyński Regional Specialist Hospital, Al. Kraśnicka 100, 20–718 Lublin, Poland; szponar.jarek@gmail.com

**Keywords:** dietary polyphenols, *Dracocephalum moldavica* L., functional food, liquid chromatography, antiradical activity

## Abstract

A new type of multigrain snack has been designed containing varied additions of Moldavian dragonhead (*Dracocephalum moldavica* L.) seeds. The antioxidant properties and the general health benefits of this plant material have already been widely acknowledged. The research discussed herein aimed to investigate the influence of the formulation and expansion method (frying) on the content of polyphenolic compounds, individual phenolic acids, and antiradical properties of innovative snacks enriched with dragonhead seeds. The highest content of polyphenols (0.685 mg GAE/mL), free phenolic acids (47.052 µg/g of dry matter), and highest radical scavenging activity (96.23% towards DPPH) were found in the fried snacks enriched with 22% of seeds. In these samples, 11 phenolic acids were detected. Strong positive correlations were seen between the addition of dragonhead and the polyphenol content (r = 0.989) and between the quantity of the enriching additive and the content of free phenolic acids (r = 0.953). The research has shown that such innovative snacks have the potential to supply health-benefiting free phenolic acids, e.g., salicylic, isoferulic, ferulic, p-coumaric, vanillic. Our studies provide an introduction to the development of a new range of functional foods.

## 1. Introduction

Snacks are considered among a wide variety of products displaying specific sensory properties. They are produced from various raw materials and with different technologies. One of the most popular groups is fried products, such as crisps and chips. Puffs are obtained from already semi-finished products subjected to the process of frying. The intermediate products, also known as pellets, are obtained from a mixture of various ingredients, starches being one of the most common. During frying, the pellets expand to give the crisps their specific structure [1]. Frying is among the most widespread forms of food processing. It is used on a massive scale in households, catering businesses, and industry. It is popular as it does not require long thermal treatment, compared to cooking or baking, and because fried products offer specific sensory properties [2]. The disadvantage of traditional deep-fat frying is excessive dehydration of the product. As a result, the product may become hard and high in fat, thus greatly increasing its energy value and becoming stodgy. A solution to this problem may be the use of pressure frying. Owing to their design, pressure fryers reduce product dehydration and excessive fat intake. Products made in such devices have a lower calorific value and demonstrate improved sensory values compared to traditional methods or frying [3,4]. Having the trend of obesity prevention in mind, the possibility of reducing the caloric value of meals by reducing the fat content at the stage of product preparation must not be ignored. Today, next to a distinctive taste and standard nutritional values, foodstuffs are expected to provide additional health benefits. This is what functional food can do, especially if enriched with biologically active secondary plant metabolites, such as polyphenols [5,6].

For this reason, the seeds of Moldavian dragonhead (*Dracocephalum moldavica* L.) deserve special attention due to their proven antioxidant properties and overall health benefits. This raw material has been tested for containing the following polyphenolic compounds: hydroxycinnamic acids (such as caffeic, coumaric, ferulic, and rosmarinic) and flavonoids [7,8]. Due to the high content of essential unsaturated fatty acids, which account for approx. 90% of all fatty acids detected in dragonhead seed oil, it is ranked among the so-called bio-oils [9]. The seeds and plant of Moldavian dragonhead are used in many regions of Asia as infusions and as a food additive, as well for the treatment of stomach and liver ailments, headache, and obstructions. The plant has been proven to alleviate the symptoms of angina, to inhibit platelet aggregation, to reduce blood viscosity, and to improve the blood supply to the heart muscle [9,10].

Despite the numerous benefits posed by the consumption of this plant, so far, dragonhead seeds have never been used as an ingredient in snacks. Our team was the first to design and produce extruded snacks enriched with dragonhead seeds. Thanks to their use, this food product could acquire health-promoting properties. Nowadays, consumers are looking for functional foods that increase the body’s natural resistance, prevent or support therapies in selected diseases, increase physical efficiency, and positively influence a person’s mental state. Therefore, the aim of our research was to design a new assortment of enriched food, which could bring additional benefits to the human body. Snack pellets (produced by the extrusion-cooking method), and fried crisps with the addition of different percentages of dragonhead seeds (0, 1, 3, 12, 22%), were tested for the presence of active compounds such as polyphenols, including free phenolic acids. The health benefits of dietary phenolic compounds depend on their bioavailability, which can be affected by differences in cell wall structure, glycoside location in cells, and especially by binding with the food matrix [2]. The frying helps to improve bioavailability by promoting chemical structural changes and the release of phytochemicals attached to the food matrix [4]. On the other hand, this high-temperature process can deactivate polyphenols contained in snack pellets. Therefore, the aim of the research was also to investigate the influence of the formulation and the expansion method (frying) on the content of investigated active compounds and the antiradical activity of food samples. Our studies provide an introduction to the development of a new range of functional foods.

## 2. Results and Discussion

### 2.1. Results for Raw Extruded Pellets

According to Navarro et al. [11], the culinary art harnessing science can significantly contribute to broader access to certain nutrients and other food components that are responsible for the salutogenic functions of food. For this reason, modern gastronomic technology, as a very practical field, should rely upon the current scientific knowledge, both food and nutrition sciences and medical sciences. Important examples of products designed based on scientific input are innovative snacks enriched with bioactive ingredients, such as the fried snacks made from extruded multigrain pellets with dragonhead seeds manufactured by our team. In addition to their primary nutritional role, they also promise additional health benefits for the body.

Multigrain pellets (half-products for expanded snacks) with different quantities of dragonhead seeds (0, 1, 3, 12, and 22%) were produced using the high-temperature short-time extrusion process. In the first stage of the research, the total content of polyphenols was measured in dragonhead seeds and in snack pellets with different quantities of the seeds. The obtained results showed that the volume of the studied compounds increased along with the addition of the seeds to the pellets. The highest content of polyphenols was found in the seeds themselves, as much as 1.308 mg GAE/mL, while the lowest was observed in snack pellets without additives, 0.235 mg GAE/mL (Table 1).

**Figure 1:**
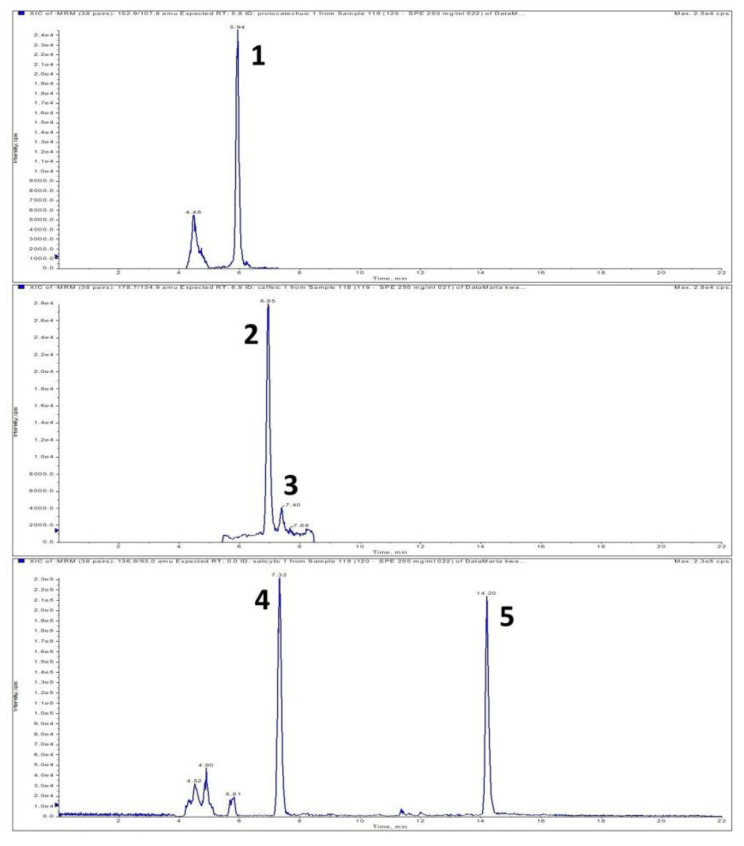

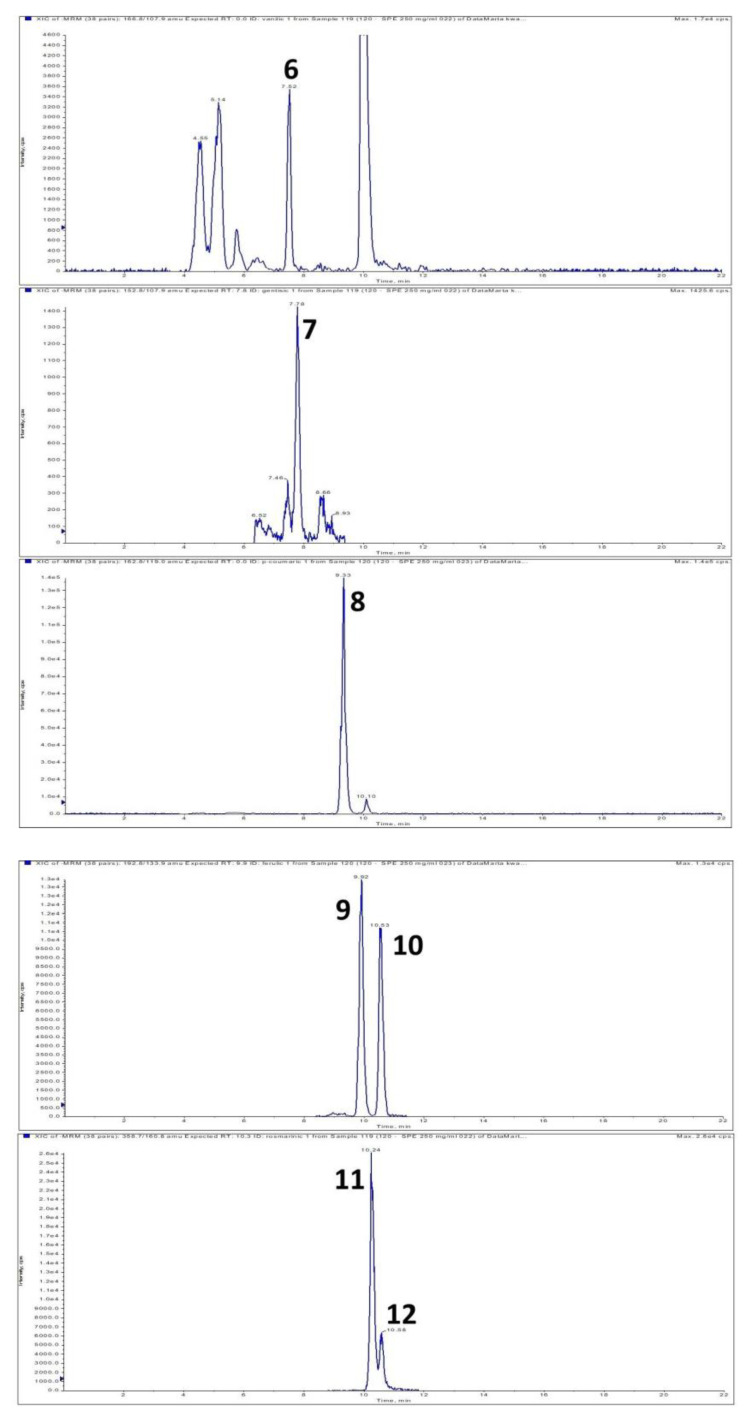
Extracted LC-MS-MRM chromatogram of phenolic acids found in snacks enriched with dragonhead seeds; MRM transitions are given in brackets: 1—protocatechuic (*m*/*z* 152.9–107.8); 2—*trans*-caffeic (*m*/*z* 178.7–134.9); 3—*cis*-caffeic (*m*/*z* 178.7–134.9); 4—4-hydroxy-benzoic (*m*/*z* 136.9—93); 5—salicylic (*m*/*z* 136.9—93); 6—vanillic acid (*m*/*z* 166.8–107.9); 7—gentisic (*m*/*z* 152.8–107.9); 8—*p*-coumaric (*m*/*z* 162.8–119); 9—ferulic (*m*/*z* 192.8–133.9); 10—isoferulic acid (*m*/*z* 192.8–133.9); 11—*trans*-rosmarinic acid (*m*/*z* 358.7–160.8); 12—*cis*-rosmarinic acid (*m*/*z* 358.7–160.8).

It was observed that health beneficial compounds from the dragonhead plant can be successfully incorporated into the pellets. During the next step, the content of free phenolic acids in the product was determined. The qualitative and quantitative analysis of phenolic acids was carried out using high-performance liquid chromatography electrospray ionization tandem mass spectrometry (LC-ESI-MS/MS). The chromatographic method was validated [12]. Ultrasound-assisted extraction (UAE) at elevated temperatures with 80% aqueous ethanol was used to obtain polyphenol extracts from the snacks. This method was selected based on previous experiments in which it was proven to be the optimum technique for isolating phenolic acids from functional foods [13]. The following phenolic acids were identified in the samples: protocatechuic, 4-hydroxybenzoic, vanillic, gentisic, salicylic (benzoic acid derivatives) and *trans*-caffeic, *p*-coumaric, ferulic, *trans*-rosmarinic, isoferulic (cinamic acid derivatives) (Figure 1, Table 2). Isoferulic acid was the prevailing phenolic acid.

As with the analysis of total polyphenols, also in this study, the content of active compounds increased as dragonhead seeds were added to the cereal pellets. The comparatively proportional increase in the content of phenolic acids demonstrates that the high-temperature and high-pressure extrusion process did not degrade the active compounds present in dragonhead seeds and in snack pellets enhanced with this raw material. The only exception was the sample with a 3% content of the seeds. It showed a lower content of gentisic, *p*-coumaric, ferulic, and isoferulic acids than in the sample with 1% addition of the raw material. This may well indicate that the ingredients had not been mixed properly during the preparation or production stage. Another reason may be concentrated overheating of the mixture in the extrusion-cooking process. It may have caused thermal degradation of the above-listed acids during the process.

Moldavian dragonhead is the source of many antioxidant molecules [7,8]. Therefore, in the next stage, the antiradical properties of this plant material and manufactured snacks were examined. The DPPH free radical scavenging potential increased along with the rising content of polyphenols and free phenolic acids in the product (Table 3).

Extrusion-cooking is the processing of starchy raw materials under specific thermal (120–200 °C), moisture, and high-pressure (20 MPa) conditions. The intense processing of mechanical shearing results in a deep transformation of individual components. This highly effective technology brings to the market new product mixes offering an attractive sensory experience. Extrusion-cooking temperature, screw speed, moisture content, feed rate, and residence time distribution are crucial for extrudate nutritional characteristics, polyphenols content, and antioxidant activity [14]. The process may deactivate antinutritional factors and enhance food antioxidant value [15]. Khanal et al. [16] reported the effects of extrusion-cooking on procyanidin monomers and dimers in grape seed and pomace. It appears to increase the levels of low-molecular-weight bioactive compounds (e.g., procyanidin) and biologically important monomers and dimers from polymer chains [16]. The intensity of these changes depends both on the properties of the raw material (e.g., humidity) and the processing parameters. A high degree of mixing and homogenization leads to a decrease in diffusion barriers and the breaking down of chemical bonds, which results in the heightened reactivity of the components. Properly selected extrusion-cooking conditions may release phenolic acids from the chemical bonds that they create with other compounds (e.g., glycosidic bonds), without deactivating aglycones [14,15,16,17]. According to the research done by Alonso et al. [17], the main factors stimulating the transformation of input material during the extrusion-cooking process are high temperatures and mechanical aspects related to shear forces, which occur along with the increase in screw rotational speed.

Many studies indicate that aglycones exhibit higher antioxidant activity than their glycosidic forms or those linked by other types of bonds [18,19,20]. The antioxidant activity of polyphenols also depends on the number of hydroxyl groups in the molecule, and it can be enhanced by spherical effects as well as synergistic and antagonistic interactions with compounds present in the matrix [20,21]. Hence, a higher content of polyphenolic compounds does not always cause improvement of antioxidant properties. Moreover, not only does the antioxidant activity of food depend on the level of bioactive compounds but it is also linked to their composition. Korus et al. [22] observed a lower antioxidant activity in dark-red kidney beans compared to black-brown and cream beans, although the dark-red bean extrudates showed a higher total phenol content compared with the black-brown and cream bean ones. In our innovative dragonhead-enriched snacks, the product’s enhanced antiradical activity occurred alongside the increase in total polyphenols and the volume of added functional ingredient. Pearson’s coefficients, exposing the relationship between the addition of dragonhead seeds, the content of polyphenols, flavonoids, and free phenolic acids, and the antiradical activity (measured after 20 min), are shown in Table 4. Very strong positive correlations were seen between the addition of dragonhead seeds and the polyphenol content (*r* = 0.998) and between the quantity of the enriching additive and the content of free phenolic acids (*r* = 0.955).

### 2.2. Results for Fried Extruded Pellets

The other and decisive stage of the research was to determine the effect of frying on the content of polyphenols, the content of individual phenolic acids, and the antiradical properties of the finished product. Frying is a pivotal factor in the technological process of snack manufacture. During frying, products acquire their most essential quality characteristics. This process also determines their health properties. Selection of proper frying fat is fundamental to a successful frying process. The fitness of frying fat for the process can be determined on the basis of its smoke point: the higher the point, the better [23]. During long-term heating of fat and the action of such factors as high temperature, atmospheric oxygen, or water contained in the fried product, fat decomposes gradually while releasing volatile (e.g., acrolein) and non-volatile compounds (e.g., hydroperoxides or acrylamide). These compounds reduce the sensory quality of fried products and are toxic to the human body [24]. The use of extra virgin oil is recommendable [25]. According to various sources, the oil smoke point ranges from 185 to 210 °C, depending on the presence of impurities and acidity. The higher the oil quality (and the lower its acidity), the higher its smoke point. However, the smoke point is not the only criterion to determine whether specific fat is suitable for frying. Refined vegetable oils recommended for frying have a high smoke point, e.g., 210–220 °C for sunflower oil. Nevertheless, they contain a large volume of chemically unstable poly-unsaturated fatty acids which oxidize at high temperatures and form compounds hazardous to the human body. Olive oil mainly contains monounsaturated oleic acid (73%): it accounts for 90% of its composition, together with other saturated acids [26]. At high temperatures, saturates and monounsaturates undergo negative alterations to a much lesser extent than sensitive polyunsaturated acids, which account for no more than 10–12% of olive oil. Olive oil abounds in polyphenols and vitamin E. They reduce the pace of harmful changes induced by high temperatures [26,27,28]. Molina-Garcia et al. [29] compared three types of fat: extra virgin olive oil, walnut oil, and rapeseed oil. The oils were heated for several hours and used for frying at various temperatures. Rapeseed oil also contains a large volume (62%) of stable monounsaturated oleic acid. However, they noticed that fewer toxic compounds were produced in the process of frying in the extra virgin olive oil, and that their formation was slower, which could be attributed to the high content of antioxidants. Relying on the collected research evidence, the authors used extra virgin olive oil to fry the pellets.

In general, total polyphenols, total free phenolic acids, as well as the antiradical properties of the finished products exhibited a positive correlation with the addition of dragonhead seeds to the fried snacks (Table 4), which was also the case with the snack pellets. In most cases, total polyphenols, total free phenolic acids, and the DPPH free radical scavenging potential showed lower values for the fried snacks than for the corresponding samples not subjected to frying. Samples with the maximum dragonhead seed content (22%) were the exception. An interesting phenomenon was observed. Two acids, vanillic and *cis*-rosmarinic, were confirmed to be present in the seeds and in fried snacks enriched with 22% of seeds, but they were missing from snack pellets. In addition, the content of the several acids—protocatechuic, *trans*-caffeic, gentisic, salicylic, and isoferulic—in fried snacks enriched with 22% of dragonhead seeds was greater than in the corresponding pellets. Total polyphenols were also significantly higher compared with the corresponding extrudates not subjected to frying.

The polyphenol profile and the antioxidant capacity evolve during the preparation of products for consumption. It is commonly believed that the effects of high-temperature food processing are destructive. Indeed, hydrothermal processes (e.g., cooking) certainly have an adverse effect on the content of water-soluble antioxidants such as polyphenols [30]. Contrasting effects are seen when using non-polar media processes, such as deep-frying. Moreover, this technique has a much weaker effect on the concentration of phenolic compounds in the plant matrix than cooking [31]. The increase in the concentration of some free phenolic acids, as noted in fried snacks compared with the raw ones, enriched with the addition of 22% of dragonhead seeds, may be the outcome of the simultaneous operation of several mechanisms. Some authors have described the transfer to the foodstuff of the phenols present in the absorbed olive oil, the effect of concentration in the food matrix after partial evaporation of moisture, and the lack of diffusion to the olive because migration of hydrosoluble substances toward polar media does not occur spontaneously [28,30]. It has been shown that there is an increase in the availability of phenols physically and chemically linked to the microstructure of the processed vegetables in comparison to the raw, whether because of the breakage or softening of the rigid cell walls and other components of the vegetable cells (vacuoles and apoplasts), or because of the decomposition of phenolic compounds linked to the fiber (cellulose and pectin) [28]. The breakdown of phenol–sugar glycosidic links giving rise to aglycons also contributes to the increase in phenol concentration [28,30]. Moreover, Martínez-Huélamo and co-workers [32,33], in a study of the effect of oil addition and processing time on the phenolic profile of tomato sauce, found an increase in polyphenol content (naringenin) when olive oil was used for processing. As mentioned above, frying increases the availability of phenols, previously bound physically and chemically. This is attributed to the cracking or softening of the rigid cell walls of the seeds and other plant cell components or to the decomposition of phenolic compounds bound with the fiber [28]. The breakdown of the glycosidic phenol–sugar bonds, which leads to the formation of aglycons, is also conducive to an increased antiradical capacity of fried snacks [30]. In our case, factors resulting from changes to the matrix may also be relevant. There is a process of gradual replacement of wheat flour with dragonhead seeds that become the main source of polyphenols in the product. In snacks with the maximum amount of functional raw material added, dragonhead seeds account for almost a quarter of the total weight of the snack.

The fried snacks enriched with 22% of dragonhead seeds showed higher antiradical properties than snack pellets. Ramírez-Anaya et al. [30] noted that the antiradical capacity of the foods processed in oil increased more than when processed in water. They observed a consistent additional effect in the ABTS results of eggplant, tomato, and pumpkin and the DPPH results of eggplant and potato. Significant increases in the antiradical capacity were found by Bellail et al. [34] using DPPH to test raw and deep-fried potatoes. The same was true for Miglio et al. [31] using ABTS for examination of fresh and sautéed carrots and zucchini. Increases were also found using the FRAP method by Dini et al. [35] in fried pumpkin. The increase in the antioxidant capacity of the processed food can be attributed to the increase in phenol concentration due to both higher availability and also to the increase associated with olive oil absorption [36,37,38]. This is supported by the fact that the total phenol concentration and the antiradical capacity showed a significant correlation (Table 4).

## 3. Materials and Methods

### 3.1. Chemicals

LC-MS-grade acetonitrile, methanol, and formic acid, analytical-grade ethanol for extraction, and Folin–Ciocalteu reagent were purchased from J.T. Baker (Phillipsburg, PA, USA). The HPLC-grade standards (gallic, protocatechuic, 4-hydroxybenzoic, vanillic, gentisic, salicylic, *trans*-caffeic, *p*-coumaric, ferulic, *cis*- and *trans*-rosmarinic, isoferulic acids) and DPPH (2,2-diphenyl-1-picrylhydrazyl) were provided by Sigma Aldrich (Sigma Aldrich, St. Louis, MO, USA). LC-MS-grade water was prepared using a MilliQ system (Millipore S.A., Molsheim, France).

### 3.2. Production of Extruded Pellets

The snack pellets were produced at the Department of Process Engineering, the University of Life Sciences in Lublin. The basic recipe for multigrain pellets was composed of: wheat flour—30.00%, wholegrain rice flour—20.50%, white Masa Harina corn flour—17.00%, wheat starch—12.00%, spelt flour—6.00%, refined rice flour—3.50%, quinoa—3.00%, oat flour—3.00%, salt and sugar—a total of 4.00%. The mixture was enriched with 1, 3, 12, and 22% addition of dragonhead as a replacement for wheat flour content. The mixture was moistened up to 33% and then was subjected to the extrusion-cooking process (temperature of the extruder head: 100 °C, screw rotation speed: 40 rpm). Snack pellets were cut into 3 cm × 3 cm squares and dried at 40 °C for 10 h.

### 3.3. Preparation of Extracts

All extrudates and dragonhead seeds were tested for their antiradical activity and the content of biologically active ingredients (total polyphenolic content and the presence of free phenolic acids). For this purpose, their extracts were prepared. The extraction process was performed in an ultrasonic bath (Bandelin Electronic GmbH & Co. KG, Berlin, Germany) with 40 mL of 80% ethanol for 40 min at a temperature of 60 °C (ultrasound frequency of 33 kHz and a power of 320 W). The extracts were filtered, and a new portion of 80% ethanol was added to the remainder to repeat the extraction. The obtained extracts were combined, evaporated to dryness, and dissolved in methanol [13].

### 3.4. Determination of the Total Content of Polyphenolic Compounds (TPC)

The total content of polyphenolic compounds was resolved utilizing the modified Folin–Ciocalteu (FC) method [30]. The amount of phenolics was expressed as mg gallic acid equivalents (GAE) per g of dry mass (d.m.).

### 3.5. Ability to Scavenge DPPH

Measurement of antiradical activity was carried out via DPPH stable radical (2,2-diphenyl-1-picrylhydrazyl) spectroscopy according to the modified method of Burda and Oleszek [39]. Absorbance was measured at 517-nm wavelength, and the UV–VIS spectrophotometer (Genesys UV-VIS, Thermo Scientific, Waltham, MA, US) was calibrated to pure methanol. The measurements were carried out every 5 min for 20 min. This approach allows the monitoring of changes in absorbance over time and indicates when the plateau is reached. Based on the results, the free radical scavenging ability of the tested extracts was calculated using the following formula:

%RSA = [(A_0_ − A_1_)/A_0_] × 100 (1)

where A_0_—the absorbance of the sample except tested extracts; A_1_—the absorbance of the sample with tested extracts.

### 3.6. Liquid Chromatography/Electrospray Ionization Triple Quadrupole Mass Spectrometry (LC-ESI-MS/MS)

Identification and quantification of phenolic acids was performed using liquid chromatography–mass spectrometry (LC-ESI-MS/MS). An Agilent 1200 Series HPLC system (Agilent Technologies, Santa Clara, CA, USA) coupled to a 3200 QTRAP Mass spectrometer (AB Sciex, Redwood City, CA, USA) and equipped with Turbo V™ source working in negative electrospray ionization mode (ESI) was used. All devices were controlled with Analyst 1.5 software (AB Sciex). Analytes were separated using an Eclipse XDB-C18 column (4.6 mm × 150 mm, 5-μm particle size; Agilent Technologies, Santa Clara, CA, USA). QTRAP worked in the multiple reaction monitoring (MRM) scan mode. All the conditions, including the LC parameters, mass spectrometer settings, and full validation protocol, were previously reported by Olech et al. (2020) [12]. Phenolic acids were identified on the basis of their retention time and MRM transitions after comparing the results with the data obtained for corresponding analytical standards. For quantitative analysis, the most intense MRM transitions and data from the corresponding calibration curves were employed.

### 3.7. Frying of Extruded Pellets

The snack pellets were deep-fried in a pressure fryer by ADA-SCE 12 (Ada Gastrogaz, Sulechów, Poland) for 15 s at 160 °C with extra virgin olive oil (commercial product purchased at a local supermarket). Ready-to-eat snacks were extracted using the same method as for snack pellets (Section 3.3). In the extracts, total polyphenolic content, ability to scavenge DPPH, and quantification of phenolic acids were determined.

### 3.8. Statistical Analysis

All the measurements were done in three replications; results were mean values of multiple repetitions ± standard deviation (SD). Statistical analysis with ANOVA (Statistica 13.0, StatSoft Inc., Tulsa, OK, USA) was used to determine the significance of differences at α = 0.05, with Duncan’s test applied to evaluate the homogenous groups. Pearson’s correlation coefficients and their significance were evaluated at 0.05 and 0.01 for the tested characteristics.

## 4. Conclusions

A shift towards a healthy lifestyle and the prevention of obesity and other diseases, such as cancer, diabetes, or neurodegenerative and cardiovascular conditions have contributed to the emergence and advancement of new nutritional trends. The current research describes studies of newly developed innovative snacks enriched with dragonhead seeds. The influence of their composition and frying procedure on the content of polyphenolic compounds, individual phenolic acids, and antiradical properties was investigated. The highest content of polyphenols and free phenolic acids and the highest radical scavenging activity were found in the fried snacks enriched with 22% of seeds. Total polyphenols, total phenolic acids, and antiradical properties of the finished products exhibited a positive correlation with the addition of dragonhead to the snacks.

Our research has shown that innovative snacks enhanced with dragonhead seeds have potential as a significant source of natural antioxidants, thus being capable of improving the health and quality of life of individuals suffering from lifestyle diseases. The presented results are a major step towards making the extruded snacks obtained in our experiments a much-demanded product ranked among functional foods.

## 5. Patents

Patent Poland, no. 228731. Snacks and the method of producing snacks. University of Life Sciences in Lublin, Lublin, PL. Authors: Agnieszka Wójtowicz, Tomasz Oniszczuk, Leszek Mościcki, Stanisław Juśko, Anna Oniszczuk

## Figures and Tables

**Table 1:** Total content of polyphenolic compounds (TPC) in snack samples depends on dragonhead seed addition and thermal treatment (*n* = 3 mean ± SD).

Addition of Dragonhead Seeds (%)	Total Content of Polyphenolic Compounds TPC (mg GAE/g s.m.)	
	Snack Pellets	Fried Snacks
0%	0.470 ^a^ ± 0.004	0.239 ^a^ ± 0.002
1%	0.546 ^ab^ ± 0.024	0.288 ^b^ ± 0.000
3%	0.560 ^b^ ± 0.016	0.554 ^c^ ± 0.024
12%	0.892 ^c^ ± 0.012	0.874 ^d^ ± 0.018
22%	1.208 ^d^ ± 0.054	1.370 ^e^ ± 0.016
Seeds	2.616 ± 0.156	

^a–e^—means indicated with similar letters in columns do not differ significantly at α = 0.05.

**Table 2:** Content of phenolic acids in snack samples enriched with dragonhead seeds and in dragonhead seeds (*n* = 3; mean ± SD).

	Addition of Dragonhead Seeds (%)	Content of Phenolic Acids (µg/g)											
		Protocatechuic	*trans*-Caffeic	4-OH-benzoic	Vanillic	Gentisic	*p*-Coumaric	Ferulic	*trans*-Rosmarinic	*cis*-Rosmarinic	Isoferulic	Salicylic	Sum
**Seeds**		2.141 ± 0.038	2.542 ± 0.105	6.327 ± 0.203	3.484 ± 0.134	0.026 ± 0.000	6.125 ± 0.325	3.582 ± 0.087	2.952 ± 0.132	0.956 ± 0.041	18.086 ± 0.248	4.163 ± 0.029	50.384
**Snack pellets**	0%	0.088 ^a^ ± 0.002	ND	0.322 ^a^ ± 0.005	ND	0.025 ^a^ ± 0.001	0.712 ^b^ ± 0.028	0.611^a^ ± 0.007	ND	ND	14.200 ^a^ ± 0.407	0.608 ^a^ ± 0.002	16.566
	1%	0.139 ^b^ ± 0.005	BQL	0.624 ^b^ ± 0.026	ND	0.029 ^b^ ± 0.001	0.992 ^c^ ± 0.003	0.993 ^c^ ± 0.004	ND	ND	18.040 ^b^ ± 0.292	0.828 ^b^ ± 0.003	21.645
	3%	0.168 ^c^ ± 0.0001	BQL	0.821 ^c^ ± 0.041	ND	0.025 ^a^ ± 0.000	0.624 ^a^ ± 0.011	0.648 ^b^ ± 0.005	ND	ND	18.162 ^b^ ± 0.327	1.124 ^c^ ± 0.025	21.572
	12%	0.432 ^d^ ± 0.012	0.168^a^ ± 0.010	1.543 ^d^ ± 0.073	ND	0.029 ^b^ ± 0.001	2.214 ^d^ ± 0.115	2.036 ^d^ ± 0.032	0.500 ^a^ ± 0.000	BQL	19.124 ^c^ ± 0.154	1.296 ^d^ ± 0.005	27.342
	22%	0.544 ^e^ ± 0.025	0.524 ^b^ ± 0.026	2.336 ^e^ ± 0.023	ND	0.032 ^b^ ± 0.002	2.772 ^e^ ± 0.025	2.288 ^e^ ± 0.004	0.583 ^b^ ± 0.021	BQL	21.442 ^d^ ± 0.348	1.326 ^e^ ± 0.008	31.847
**Fried** **snacks**	0%	0.092 ^a^ ± 0.002	ND	0.528 ^a^ ± 0.000	ND	0.023 ^b^ ± 0.000	0.316 ^a^ ± 0.005	0.384 ^a^ ± 0.007	ND	ND	14.400 ^a^ ± 0.701	0.608 ^a^ ± 0.003	16.351
	1%	0.106 ^b^ ± 0.005	ND	0.532 ^a^ ± 0.014	ND	0.023 ^b^ ± 0.001	0.444 ^b^ ± 0.003	0.395 ^a^ ± 0.002	ND	ND	15.920 ^b^ ± 0.052	0.608 ^a^ ± 0.002	18.028
	3%	0.113 ^b^ ± 0.000	ND	0.643 ^b^ ± 0.031	ND	0.016 ^a^ ± 0.000	0.484 ^c^ ± 0.003	0.505 ^b^ ± 0.012	ND	ND	19.240 ^c^ ± 0.302	0.620 ^a^ ± 0.021	21.621
	12%	0.408 ^c^ ± 0.016	BQL	1.012 ^c^ ± 0.048	1.808 ^a^ ± 0.009	0.032 ^c^ ± 0.001	0.968 ^d^ ± 0.022	0.656 ^c^ ± 0.024	0.584 ^a^ ± 0.015	BQL	19.320 ^c^ ± 0.424	2.392 ^b^ ± 0.040	27.180
	22%	0.616 ^d^ ± 0.023	0.760 ± 0.032	2.046 ^d^ ± 0.101	2.672 ^b^ ± 0.035	0.038 ^d^ ± 0.002	1.256 ^e^ ± 0.043	1.204 ^d^ ± 0.055	0.596 ^a^ ± 0.002	0.382 ± 0.013	35.082 ^d^ ± 0.882	2.400 ^b^ ± 0.111	47.052

BQL—results above limit of detection and below limit of quantitation, ND—not detected. ^a–e^—means indicated with similar letters in columns do not differ significantly at α = 0.05; analysis was done separately for snack pellets and fried snacks.

**Table 3:** Radical scavenging activity (%) of snack samples depends on time and dragonhead seed addition (*n* = 3 mean ± SD).

Radical Scavenging Towards DPPH (%)											
Time (min)	Addition of Dragonhead Seeds (%)										
	Seeds	Snack Pellets					Fried Snacks				
		0%	1%	3%	12%	22%	0%	1%	3%	12%	22%
**0**	26.44 ± 0.151	18.25 ^b^ ± 0.212	19.57 ^c^ ± 0.051	19.69 ^c^ ± 0.110	17.92 ^a^ ± 0.062	22.18 ^d^ ± 0.683	13.25 ^a^ ± 0.212	13.54 ^a^ ± 0.105	14.19 ^b^ ± 0.120	15.21 ^c^ ± 0.064	20.17 ^d^ ± 0.163
**5**	87.23 ± 1.210	62.77 ^a^ ± 1.330	64.90 ^b^ ± 0.421	77.83 ^c^ ± 0.210	80.89 ^d^ ± 0.897	83.02 ^e^ ± 0.501	60.35 ^a^ ± 0.063	68.07 ^c^ ± 0.389	62.87 ^b^ ± 2.315	74.89 ^d^ ± 0.897	82.75 ^e^ ± 0.933
**20**	97.23 ± 0.041	85.13 ^a^ ± 2.198	88.55 ^b^ ± 0.200	91.35 ^c^ ± 0.801	92.95 ^c^ ± 2.291	94.55 ^c^ ± 1.302	83.99 ^a^ ± 0.198	87.19 ^b^ ± 1.983	90.92 ^c^ ± 0.008	91.01 ^c^ ± 1.930	96.23 ^d^ ± 1.323

^a^^–e^—means indicated with similar letters in rows do not differ significantly at α = 0.05; analysis was done separately for snack pellets and fried snacks.

**Table 4:** Pearson’s correlation coefficients for snacks supplemented with dragonhead seeds.

	Total Polyphenols	Free Phenolic Acid	DPPH Radical Scavenging Activity
**Snack Pellets**			
Dragonhead seed content	0.998 **	0.955 **	0.852 *
Total polyphenols		0.966 **	0.856 *
Free phenolic acids			0.940 **
**Fried snacks**			
Dragonhead seed content	0.989 **	0.953 **	0.900 **
Total polyphenols		0.933 **	0.993 **
Free phenolic acids			0.767 *

*—significant at 0.05; **—significant at 0.01.

## Sample Availability

Samples of the snacks are available from the authors.
